# Application of Forced Oscillation Technique in Assessing Pulmonary Fibrosis in Hermansky–Pudlak Syndrome

**DOI:** 10.3390/arm92060040

**Published:** 2024-10-24

**Authors:** Wilfredo De Jesús-Rojas, Luis Reyes-Peña, José Muñiz-Hernandez, Rolando Mena-Ventura, Gabriel Camareno-Soto, Gabriel Rosario-Ortiz, Marcos J. Ramos-Benitez, Monica Egozcue-Dionisi, Enid Rivera-Jimenez, Rosa Román-Carlo

**Affiliations:** 1 Department of Pediatrics and Basic Science, Ponce Health Sciences University, Ponce, PR 00716, USA; grosario@psm.edu (G.R.-O.); mjramos@psm.edu (M.J.R.-B.); phc112n@gmail.com (M.E.-D.); r.romancarlofccp@gmail.com (R.R.-C.); 2 Department of Medicine, San Juan Bautista School of Medicine, Caguas, PR 00725, USA; luisrp@sanjuanbautista.edu (L.R.-P.); josemuniz4510@gmail.com (J.M.-H.); 3 Department of Medicine, Universidad Central del Caribe, Bayamón, PR 00960, USA; rolomena5@gmail.com (R.M.-V.); 121gcamareno@uccaribe.edu (G.C.-S.); 4 Department of Pediatrics, University of Puerto Rico, Medical Sciences Campus, San Juan, PR 00921, USA; enid.rivera6@upr.edu

**Keywords:** forced oscillation technique, pulmonary fibrosis, Hermansky–Pudlak syndrome, respiratory mechanics

## Abstract

**Highlights:**

**What are the main findings?**

- Progressive changes in pulmonary resistance and reactance measure by the forced oscillation technique (FOT) were seen in patients with Hermansky–Pudlak syndrome (HPS) at different severities of pulmonary fibrosis.

**What is the implication of the main finding?**

- Respiratory impedance (Zrs) measured by the FOT is a valuable tool for detecting and monitoring the progression of pulmonary fibrosis in patients with HPS with the HPS-1 (c.1472_1487dup p.His497Glnfs*90) founder mutation.

**Abstract:**

Hermansky–Pudlak syndrome (HPS) is a rare autosomal recessive disorder characterized by defects in lysosome-related organelles. Given the high mortality rate associated with HPS pulmonary fibrosis (PF) and the significant risks tied to lung transplantation, it is essential to explore new tools for the early surveillance of PF to monitor its progression before clinical symptoms become apparent. This study evaluates the forced oscillation technique (FOT) for assessing PF in five adult patients with HPS, all homozygous for the *HPS-1* (c.1472_1487dup p.His497Glnfs*90) founder mutation. Using the Resmon™ Pro V3 device, the FOT measured resistance (Rrs) and reactance (Xrs) at 5, 11, and 19 Hertz (Hz). High-resolution computed tomography (HRCT) scans of the chest were reviewed for radiographic findings. The cohort (*n* = 5) had a median age of 43 years. All patients exhibited HPS clinical features, including oculocutaneous albinism and respiratory symptoms such as dry cough and dyspnea. Radiographic analysis revealed PF in four patients (80%), with traction bronchiectasis, reticular patterns, honeycombing, and ground-glass opacities. The FOT detected progressive changes in pulmonary resistance and reactance correlating with fibrosis severity. These findings suggest that the FOT is a valuable non-invasive tool for monitoring PF in patients with HPS-1, potentially improving early diagnosis and management.

## 1. Introduction

Hermansky–Pudlak syndrome (HPS) is a rare autosomal recessive disorder characterized by defects in the formation of lysosome-related organelles, such as melanosomes in melanocytes, δ-granules in platelets, and lamellar bodies in type II pneumocytes [1]. These organelles are crucial for the production of melanin, the aggregation of platelets for blood clotting, and the reduction in alveolar surface tension, respectively [2]. Consequently, the clinical features of HPS include oculocutaneous albinism, bleeding susceptibility, and pulmonary fibrosis, particularly in subtypes 1, 2, and 4 [3]. Patients with HPS who develop pulmonary fibrosis often present with progressive dyspnea upon exertion and chronic cough [4]. The disease typically progresses to the point where lung transplantation or death from respiratory failure becomes unavoidable [3,4]. Given the high mortality rate associated with HPS pulmonary fibrosis and the significant risks tied to lung transplantation, it is essential to explore new tools for the early surveillance of pulmonary fibrosis to monitor its progression before clinical symptoms become apparent.

The forced oscillation technique (FOT) has shown promise for patients with severely impaired lung function who may have difficulties in performing spirometry due to poor effort or other limitations [5,6]. The FOT utilizes external pulse vibrations directed at the mouth to measure the flow response of the respiratory tract during normal breathing, making it effective, even in patients with severe pulmonary disease [6,7]. Specifically, the FOT provides measurements of respiratory impedance (Zrs), which can be divided into resistance (Rrs) and reactance (Xrs) [8]. Rrs represents the energy required to move the pressure wave through the airways and tends to increase in diseases such as asthma or chronic obstructive pulmonary disease. Xrs, on the other hand, reflects the elastic properties of lung tissue and interstitial forces, with more negative values indicating reduced elasticity as seen in interstitial lung diseases like pulmonary fibrosis. Recent studies have demonstrated that the FOT can serve as an early screening tool to detect proximal and distal pulmonary tissue damage, even before significant changes are observed in spirometry results [9]. This technique is particularly useful for assessing airflow limitations and monitoring chronic disease progression in populations such as children, the elderly, and those unable to perform spirometry [10]. Despite its potential, there is no information on the use of the FOT in assessing PF in patients with HPS. Given the increased mortality risk associated with the presence of pulmonary fibrosis in HPS within a decade [3], it is crucial to establish appropriate lung surveillance protocols to track changes in pulmonary function over time clinically and to monitor drug response in future clinical trials. Therefore, this study aims to explore the application of the FOT as a non-invasive method for assessing pulmonary Rrs and Xrs in patients with HPS pulmonary fibrosis at various stages of the disease.

## 2. Materials and Methods

A retrospective chart review was performed on five adult patients (n = 5) with HPS, all homozygous for the *HPS-1* (c.1472_1487dup p.His497Glnfs*90) founder mutation [11], who were seen at the HPS multidisciplinary clinic in Mayaguez, Puerto Rico, which is at the west side of the island. Spirometry parameters, including forced vital capacity (FVC), forced expiratory volume in one second (FEV1), the FEV1/FVC ratio, forced expiratory flow at 25–75% of the pulmonary volume (FEF 25–75%), and maximum forced expiratory flow (FEF max), were evaluated following the American Thoracic Society (ATS) guidelines in a certified clinical setting [12]. Airflow limitation was assessed using Z-score values, with −1.64 serving as the lower limit of normal, based on the Global Lung Function Initiative-2012 (GLI) reference equation [13]. The forced oscillation technique (FOT) was utilized at baseline using the Resmon™ Pro V3 device (RESTECH Srl, Milano, Italy) to measure resistance [cmH_2_O/(s/L)] and reactance [cmH_2_O/(s/L)] [14]. For pediatric patients, the FOT was conducted using 10 acceptable breaths at 8 Hz, while a multi-frequency mode of 5–11–19 Hz was used for adult patients, in accordance with European Respiratory Society (ERS) standards. In addition to evaluating respiratory impedance with the FOT, high-resolution computed tomography (HRCT) scans of the chest were retrospectively analyzed to assess the radiographic findings associated with pulmonary fibrosis in patients with HPS.

## 3. Results

This study included a cohort of five patients with HPS homozygous for the Puerto Rican HPS-1 (c.1472_1487dup p.His497Glnfs*90) founder mutation. The median age of the HPS patients was 43 years, with the cohort comprising three males and two females, all of Hispanic ethnicity (Puerto Rican). All patients (five out of five, 100%) exhibited the clinical characteristics of HPS, including oculocutaneous albinism, bleeding susceptibility, and nystagmus. Respiratory symptoms reported by the patients included dry cough, shortness of breath, and fatigue during activities, affecting almost all participants (four out of five, 80%). Table 1 provides a comprehensive summary of the demographic data, detailing the age, gender, ethnicity, and clinical characteristics of the study participants.

Table 2 presents the baseline spirometry results of five patients with HPS due to the (c.1472_1487dup p.His497Glnfs*90) founder mutation. The spirometry values were abnormal in most of our cohort (4/5, 80%) and exhibited a restrictive pattern of airflow at different severity levels of pulmonary fibrosis. FVC values ranged from 68% to 38% of the predicted values. FEV1 values ranged from 70% to 39% of the predicted values. The FEV1/FVC ratio ranged from 105% to 92% of the predicted values, all consistent with a restrictive pattern of airflow. The FEF 25–75% values ranged from 98% to 37% of the predicted values. The FEF max values ranged from 112% to 56% of the predicted values.

**Figure 1:**
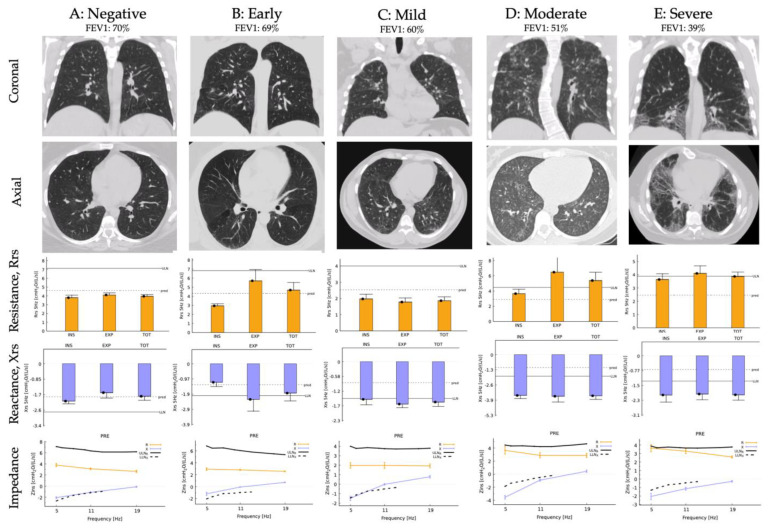
HRCT scans of the chest from five Puerto Rican patients with HPS-1, all carrying the same pathogenic founder mutation (HPS-1, c.1472_1487dup p.His497Glnfs*90). The figure is divided into sections: coronal and axial views illustrate the structural details of the chest. The graphics detail pulmonary resistance (Rrs) and reactance (Xrs), measured via the forced oscillation technique (FOT), against the severity of pulmonary fibrosis, highlighting the functional impact of the disease on lung mechanics. The pulmonary impedance was measured at frequencies of 5, 11, and 19 Hertz (Hz). The bold line in the resistance section indicates the upper limit of normal for resistance (ULN_R_), while the bold line in the reactance section delineates the lower limit of normal for reactance (LLN_X_), providing a visual representation of deviations from normal pulmonary function in these patients. FEV1: forced expired volume in 1 s.

Figure 1 presents the HRCT scans of the chest from patients and their respective FOT measurements. Case A: A patient negative for pulmonary fibrosis on HRCT, homozygous for the HPS-1 mutation, had an FEV1 of 70% of the predicted value. The patient demonstrated normal Rrs and Xrs values that were below the upper limit of normal for resistance (ULN_R_) and above the lower limit of normal for reactance (LLN_X_). Case B: a patient presented with early signs of pulmonary fibrosis and an FEV1 of 69% demonstrated normal Rrs values (Rrs < ULN_R_) but increasingly negative expiratory Xrs values (Xrs < LLN_X_). Case C: a patient presented with mild pulmonary fibrosis and an FEV1 of 60% with abnormal Xrs (Rrs < ULN_R_ and Xrs < LLN_X_). Case D: a patient with moderate pulmonary fibrosis and an FEV1 of 51% produced abnormal values for both Rrs and Xrs (Rrs > ULN_R_ and Xrs < LLN_X_). Case E: a patient with severe pulmonary fibrosis and an FEV1 of 39% also presented with values that exceeded the normal limits of Rrs and Xrs (Rrs > ULN_R_ and Xrs < LLN_X_).

## 4. Discussion

The findings from this study provide evidence for the clinical relevance of the forced oscillation technique (FOT), demonstrating its ability to capture changes in respiratory impedance that correlate with the progression of pulmonary fibrosis in patients with HPS. Pulmonary fibrosis occurs in patients with HPS with subtypes 1, 2, and 4, typically presenting between 30 and 40 or 50 and 60 years of age [1,3,4]. HPS pulmonary fibrosis exhibits histological and clinical patterns similar to idiopathic pulmonary fibrosis (IPF), presenting with dyspnea, cough, and progressively worsening hypoxemia [1,3,4]. The exact pathogenesis of HPS-PF remains unclear; however, it is characterized by the progressive and irreversible scarring of lung tissue, ultimately leading to respiratory failure and death within approximately 10 years of onset [1,2,3,4]. Diagnosis is made with HRCT scans of the chest [1,4]. HRCT also allows for the evaluation of HPS pulmonary fibrosis progression, which is characterized by ground-glass opacities, a reticulation of interstitial spaces, and, in advanced disease, the loss of lung volume, honeycombing, and traction bronchiectasis [1,3,4]. Clinical management for patients with HPS at risk for HPS pulmonary fibrosis involves preventive care for asymptomatic individuals, supportive care for symptomatic patients, and lung transplantation for those with severe disease [4]. Currently, there are no medications approved for the treatment of HPS pulmonary fibrosis; thus, lung transplantation remains the only effective treatment [1,3]. However, lung transplantation is a complex process involving the identification of a suitable donor and the execution of a high-risk procedure [1]. Additionally, lung transplant centers are limited across the U.S., and there are currently no lung transplant centers available in Puerto Rico, where HPS-1 is most frequently reported [1].

In addition to HRCT scans of the chest, annual pulmonary function testing is recommended for the surveillance of pulmonary fibrosis in patients with HPS at risk [15]. Lung function values in HPS pulmonary fibrosis typically correlate with the severity of the disease, although patients with early-stage pulmonary fibrosis may present with normal values [4]. Spirometry results in symptomatic patients may show a similarly reduced forced vital capacity (FVC), forced expiratory volume in 1 s (FEV1), total lung capacity (TLC), vital capacity (VC), and diffusing capacity of the lung for carbon monoxide (D_LCO_) [4]. As seen in Table 2, all subjects exhibited a restrictive airflow pattern of lung disease, demonstrating a reduced FEV1 and FVC and normal FEV1/FVC ratio [4]. Additionally, the degree of pulmonary function reduction in the moderate and severe cases (D and E) of HPS pulmonary fibrosis was much larger than what was seen in the negative and early cases (A and B), consistent with previous studies that demonstrated a reduction in pulmonary function testing as the disease burden advanced.

Pulmonary physiology is commonly evaluated through spirometry in clinical practice; however, spirometry is poorly sensitive in the early stages of lung function abnormalities and requires breathing maneuvers that can be challenging for children, elderly patients and those with motor or cognitive impairment [5,6]. Alternatively, the FOT is a non-invasive approach that has been shown to identify lung mechanic changes in the initial stages of respiratory pathology, even in the presence of normal spirometry results [9]. Various studies have demonstrated the utility of the FOT in managing respiratory diseases, including chronic obstructive pulmonary disease (COPD), asthma, cystic fibrosis, and interstitial lung disease [5,6,16,17]. However, there have been no studies investigating the use of the FOT in patients with HPS, making this the first application of this technique on this population. Therefore, the results of this study could have important implications for the diagnosis, monitoring, and clinical management of pulmonary fibrosis in patients with HPS. Further investigations and analysis are essential to establish guidelines in the management of patients with HPS to maximize their functionality and improve their quality of life.

The FOT uses sound waves of single frequencies that are transmitted into the lungs during tidal breathing [9,10]. These sound waves cause changes in pressure and, subsequently, changes in airflow, which can be measured to determine the mechanical properties of the lung [9]. The output consists of the respiratory impedance (Zrs), which includes the respiratory resistance (Rrs) and respiratory reactance (Xrs) over a range of frequencies (classically from 3 to 35 Hz) [10]. The use of multiple oscillation frequencies permits the differentiation of large airway behavior from that of the peripheral small airways [10]. Rrs reflects the resistance of the oropharynx, larynx, trachea, large and small airways, lung, and chest wall tissue [10]. Xrs consists of the mass inertive forces of the moving air and the elastic properties or compliance of the lung periphery [10]. Studies have found that in both obstructive and restrictive lung diseases, there is an increase in Rrs and a decrease in Xrs (more negative) at 5 Hz (increased Rrs5 and decreased Xrs5) [18]. However, they can be differentiated at 20 Hz, where restrictive lung diseases demonstrate normal Rrs (normal Rrs20), and obstructive lung diseases exhibit an increased Rrs (increased Rrs20) [19].

As shown in Figure 1, all Rrs and Xrs values for our patient with HPS but no HPS pulmonary fibrosis (A) were below the upper limit of normal for resistance (ULN_R_) and above the lower limit of normal for reactance (LLN_X_). In patients with HPS who showed early and mild pulmonary fibrosis (B and C), normal values for Rrs were noted, but there was a marked negative increase in Xrs below the LLN_X_. A more negative value for Xrs indicates a decrease in lung compliance and increased stiffness of lung tissue [20]. This pattern is representative of interstitial lung disease, in this case, pulmonary fibrosis secondary to HPS [21]. In our patients with moderate and severe PF (D and E), most values exceeded the normal limits for Rrs and Xrs. The findings from the moderate and severe patients provide an insight into the behavior of PF in patients with HPS who harbor the *HPS-1* (c.1472_1487dup p.His497Glnfs*90) mutation. As the disease progresses, Rrs increases above the ULNR, making it more challenging for the pressure wave to move through the respiratory airways, probably due to the presence of traction bronchiectasis [21]. However, the elevation in Rrs, though above normal limits, remains borderline. In contrast, changes in Xrs are more pronounced and significant with disease severity, especially at lower frequencies (5 Hz). This information is crucial as it indicates that patients with HPS in our cohort exhibit the natural, pathological progression of pulmonary fibrosis characterized by increased resistance but, even more notably, reduced reactance.

Considering the spirometry values for all patients with HPS in our cohort and the convenience of a passive, non-invasive technique, the FOT emerges as an extremely useful tool for assessing pulmonary function in patients with HPS, particularly those with lower spirometry values and more developed pulmonary fibrosis [19]. Establishing standardized protocols for FOT use and interpretation in HPS will be crucial for optimizing clinical guidelines to improve diagnosis and surveillance in this patient population. Also, future studies exploring different HPS subtypes are called for to refine the FOT’s validation in HPS. Additionally, research applying the FOT in more extensive, longitudinal studies involving other interstitial lung diseases could help compare them with HPS and have a more comprehensive view of clinical outcomes for every type of interstitial lung disease.

## 5. Conclusions

This study demonstrates the potential utility of the FOT in evaluating and monitoring pulmonary fibrosis in patients with HPS. By assessing five adult patients homozygous for the *HPS-1* (c.1472_1487dup p.His497Glnfs*90) founder mutation, we observed significant changes in pulmonary resistance and reactance that correlate with the severity of fibrosis. HRCT scans of the chest corroborated these findings, revealing characteristic features of pulmonary fibrosis such as traction bronchiectasis, reticular patterns, honeycombing, and ground-glass opacities. These results highlight the potential of the FOT as a non-invasive tool for the detection and ongoing monitoring of lung function deterioration in patients with HPS. The integration of the FOT into clinical practice could significantly enhance the management of pulmonary fibrosis in HPS by providing a new method for tracking disease progression, especially for patients with poor effort on standard spirometry. This approach could lead to more timely interventions, potentially improving patient outcomes and quality of life. Future studies with larger cohorts and longitudinal designs are warranted to further validate the FOT’s sensitivity and efficacy and to explore its application across different stages of pulmonary fibrosis in HPS and other interstitial lung diseases.

## Figures and Tables

**Table 1:** Demographic data and clinical characteristics of five patients with Hermansky–Pudlak syndrome (HPS) who possess the HPS-1 (c.1472_1487dup p.His497Glnfs*90) founder mutation.

Demographic Characteristics	(*n* = 5)
Age, median ± IQR	43 ± 19.5
Gender	-
Female, n (%)	2 (40)
Male, n (%)	3 (60)
Ethnicity	-
Hispanics, Puerto Ricans, n (%)	5 (100)
*HPS-1* (c.1472_1487dup p.His497Glnfs*90), n (%)	5 (100)
HPS Pulmonary Fibrosis Signs and Symptoms:	
Dry cough, n (%)	4 (80)
Fatigue with activities, n (%)	4 (80)
Shortness of breath (dyspnea), n (%)	3 (60)
Median FEV1, median ± IQR	59 ± 23.5
Clubbing, n (%)	0 (0)
Radiographic Findings	-
Diagnosis of pulmonary fibrosis, n (%)	4 (80)
Traction bronchiectasis, n (%)	1 (20)
Reticular pattern, n (%)	3 (60)
Honeycombing, n (%)	2 (40)
Ground-glass opacities, n (%)	4 (80)

FEV1: forced expired volume in 1 s. All values are represented in percentages except for age, represented as a median.

**Table 2:** Baseline spirometry results of patients with HPS due to the (c.1472_1487dup p.His497Glnfs*90) founder mutation.

Cases	PF	Gender	Age	FVC	FEV1	FEV1/FVC	FEF 25–75	FEF Max	Airflow
A	Negative	F	36	67 *	70 *	105	82	72	Restrictive
B	Early	M	47	68 *	69 *	101	67	103	Restrictive
C	Mild	M	51	59 *	60 *	92	98	112	Restrictive
D	Moderate	F	23	50 *	51 *	105	77	87	Restrictive
E	Severe	M	43	38 *	39 *	101	37	56	Restrictive

Forced vital capacity (FVC), forced expired volume in 1 s (FEV1), FEV1-to-FVC ratio (FEV1/FVC), forced expiratory flow at 25 and 75% of the pulmonary volume (FEF 25–75%), and maximum forced expiratory flow (FEF Max) taken following the American Thoracic Society (ATS) guidelines. All values are represented in percentages predicted for age. (*) Values in red were considered abnormal if less than 80% of predicted. PF: pulmonary fibrosis. Age presented in years.

## Data Availability

All data are available upon request through the corresponding author.
